# Combined Toxic Exposures and Human Health: Biomarkers of Exposure and Effect

**DOI:** 10.3390/ijerph8030629

**Published:** 2011-02-24

**Authors:** Ilona Silins, Johan Högberg

**Affiliations:** Institute of Environmental Medicine, Karolinska Institutet, Box 210, 171 77 Stockholm, Sweden; E-Mail: ilona.silins@ki.se

**Keywords:** biomarkers, combined exposure, chemical mixture, occupational exposure

## Abstract

Procedures for risk assessment of chemical mixtures, combined and cumulative exposures are under development, but the scientific database needs considerable expansion. In particular, there is a lack of knowledge on how to monitor effects of complex exposures, and there are few reviews on biomonitoring complex exposures. In this review we summarize articles in which biomonitoring techniques have been developed and used. Most examples describe techniques for biomonitoring effects which may detect early changes induced by many chemical stressors and which have the potential to accelerate data gathering. Some emphasis is put on endocrine disrupters acting via epigenetic mechanisms and on carcinogens. Solid evidence shows that these groups of chemicals can interact and even produce synergistic effects. They may act during sensitive time windows and biomonitoring their effects in epidemiological studies is a challenging task.

## 1. General Introduction

Humans are subjected to a range of chemical exposures from the environment. Chemicals in air, water, soil and food, occupational exposures and lifestyle factors, all contribute to a complex exposure situation in our daily life. It has long been known that toxicity can be modified by simultaneous or sequential exposure to multiple agents in the environment. For some combined or mixed exposures the health effects may increase more than what would be expected from simply adding the effects of the individual components, therefore there is a concern that several less studied complex exposures may have a large impact on our health as a result of combined or mixed effects.

The development and improvement of risk assessment procedures for combined and mixed exposures is an issue of many authorities world-wide, with on-going activities in e.g., WHO, USA and the European Union. The US Environmental Protection Agency published their first guideline on risk assessment of chemical mixtures in 1986, which was subsequently supplemented in 2000 [1,2]. Other activities towards a cumulative risk assessment approach are currently in progress [3]. Within the WHO, the International Programme on Chemical Safety (IPCS) Harmonisation Project currently develop a framework for risk assessment of combined exposure to multiple chemicals [4]. Recently, the Council of the European Union emphasized the need to consider combined and mixed exposures of chemicals in future risk assessments, and to further develop legislation, methodology, and to support research within this area [5].

The need to consider a greater range of factors contributing to potential health effects of combined exposures makes the risk assessment process more complex compared to the assessment of single chemicals. For example, an increased understanding and knowledge about the individual agents, uptake, metabolism, excretion and mechanisms/modes of action in different tissues and cells as well as temporal factors are needed for evaluating health risks of combined and mixed exposures. Even though there has been recent progress within this area of research, the development of risk assessment approaches for combined exposures is still hampered by lack of data. Techniques and methods need to be further developed to fill data gaps and increase the knowledge on harmful exposure combinations.

In a recent review by Manno *et al.* biomonitoring is defined as “the repeated, controlled measurement of chemical or biological markers in fluids, tissues or other accessible samples from subjects exposed or exposed in the past or to be exposed to chemical, physical or biological risk factors in the workplace and/or the general environment” [6]. Biomonitoring can be used to discover exposures to chemicals that may cause harm to human health. Biomarker data is often critical for chemical risk assessment and we found that biomarker information of combined or mixed exposure is a particular weak point in risk assessment.

In this review, we will describe aspects of the problem posed by biomonitoring combined or mixed exposures to toxicants in humans. We will present examples showing how combined exposures have been biomonitored. Literature searches indicate that some areas have been more frequently covered than other areas, and we hope this will be reflected by our examples. The examples have been selected to illustrate biomonitoring of exposure and biomonitoring of effects at an organ level and at a cellular or subcellular level. Chemical carcinogenesis and endocrine disruptions are two areas frequently implicated in combined actions and additional examples have been taken from these areas of research. We will end this review by discussing future developments expected in this field.

## 2. Mixtures

A mixture is defined as the combination of two or more environmental agents [7]. Mixtures can be categorized in many ways, e.g., as being simple or complex [8]. Simple mixtures contain a well-defined number of components, in contrast to complex mixtures. Examples of complex mixtures found in our environment are diesel exhaust, cigarette smoke, creosote, and asphalt fumes. For most complex mixtures, the exact composition is usually not fully characterized. Some mixtures can contain hundreds of chemicals and may vary with the site of origin and the exposure situation. A mixture can be intentionally produced, generated or could arise coincidentally [9]. The generated and coincidental mixtures created in our environment are probably countless and how these impact on human health are largely unknown.

There are two main principles describing how individual chemicals in a mixture affect one another: the concept of additivity and interaction. Additivity assumes that chemicals act by the same or different modes of action, which results in dose or effect addition. Interaction assumes that individual chemicals affect toxicity of one another, either by synergism or antagonism (more or less than an additive effect) [10]. Examples of interactions are agent-to-agent interactions, toxicokinetic and toxicodynamic interactions [7,11–13]. For both the models described above there are mathematically based methods used to predict toxicity of mixtures. However, toxicity is not always simple to predict for complex mixtures. Modes of actions for some chemicals may be unknown and interacting effects may differ depending on dose and dose ratio [14]. Depending on the availability of data, different strategies for assessing the effects and risks of mixtures or combined exposures are employed for risk assessment. For example, the risk could be assessed using data of the mixture in question (whole-mixture approach), using data of a similar mixture or of individual chemicals (and employing the concept which best applies to the particular mixture) [2,15]. This seemingly simple way to categorize interactions is complicated by the fact that e.g., single carcinogens or endocrine disrupters may act in separate time windows to synergize or antagonize their effects e.g., over a lifetime. Well-studied interactions of this type are those between so called tumour initiators and promoters. More recent examples include similar types of interactions between endocrine disrupters and carcinogens [16].

## 3. Biomarkers of Exposure, Effects, and Susceptibility

Biomarkers used in human health studies are typically divided into three classes; biomarkers of exposure, effect and susceptibility. Biomarkers of exposure involve measurements of parent compound, metabolites or DNA- or protein adducts and reflect internal doses, the biologically effective dose or target dose. Biomarkers of effects could be changes on a cellular level, such as altered expression of metabolic enzymes but could also include markers for early pathological changes in complex disease developments, such as mutations and preneoplastic lesions. Sometimes the classification is overlapping, e.g., DNA adducts could be used as biomarkers of exposure but may also imply an effect [17]. Biomarkers of susceptibility indicate an often constitutive ability of an individual to respond to specific exposures.

Biomarkers of exposure are preferably specific for the chemicals of exposure, while biomarkers of effect often are unspecific for the agent in question [18]. This simple notion suggests that biomarkers of effect should have the greater potential to reflect complex exposures and should also have the ability to include aggregated and sequential exposures over time. Another comment is that the use of biomarkers of effect in studies of complex exposures could help to identify both the active components of the mixtures/combined exposure as well as the consequences of specific mixture exposures. For example, it has been convincingly shown that work as a painter is associated with increased risk of cancer. However, the specific carcinogenic agent or agents have not been characterized and further studies are needed for cancer prevention [19]. These include the biomonitoring of exposure to individual agents and the biomonitoring of early effects for identifying causative agents, or rather mixtures of agents.

An example of a biomarker of exposure that has been used in many studies on complex mixtures of polycyclic aromatic hydrocarbons (PAHs) is the excretion of 1-hydroxypyrene in urine [13]. PAHs belong to a group of chemicals formed as complex mixtures in many combustion processes. Many PAHs have been shown to be carcinogenic in animals via a genotoxic mode of action and are of great toxicological concern. Benzo(a)pyrene is perhaps the most well studied PAH and was recently classified as a human carcinogen by IARC [20]. Detecting and quantifying PAHs in air samples is a challenging task, and benzo(a)pyrene is often used as a marker for all PAHs. This approach is far from ideal as the composition may vary with the sources and with time since formation. Moreover, there are several other even more potent carcinogens than benzo(a)pyrene found in many PAH mixtures, one example is dibenzo(a,l)pyrene. It was recently shown that dibenzo(a,l)pyrene contributed more than any other PAH to the carcinogenic potency of particles in ambient Stockholm air [21]. In an effort to reduce these problems, many studies have employed 1-hydroxypyrene excretion in urine as a biomarker for PAH exposure. Pyrene is one of the most abundant hydrocarbons in PAH mixtures and is considered a more sensitive biomarker than benzo(a)pyrene. Biomonitoring of 1-hydroxypyrene in urine has e.g., been used in studies of people working in aluminium smelter plants and in workers exposed to asphalt fumes or creosote [22–24]. Recently metabolites of another PAH, phenanthrene, has been used as a biomarker for occupational exposure of PAH [25]. A method for measuring several volatile organic compounds (VOCs) in alveolar air from exposed workers was recently suggested [26]. This method allowed biomonitoring up to 26 single VOCs and this method could be used for measuring biomarkers of exposure in workplaces with complex VOC exposure.

DNA adducts are often considered biomarkers of exposure, whereas gene mutations and chromosomal alterations are often considered biomarkers of early biological effects in carcinogenic processes [27]. Other examples of biomarkers of effect of relevance for complex mixtures are measurements of changes in biological systems, e.g., acetylcholinesterase inhibition by mixtures or combined exposure to organophosphate pesticides [28].

Proteomics and toxicogenomics techniques could help to discover new biomarkers of effect. For example, altered gene expression patterns were studied in cells exposed to an urban dust particulate complex mixture, and these data may be used to develop new techniques for biomonitoring effects of urban dust. In further support for the potential importance of this method for biomonitoring of mixtures or combined exposures, global analyses of gene expression data demonstrated changes in more than 40 RNA transcripts in response to the mixed exposure [29].

Biomarkers of susceptibility may include polymorphisms of specific genes associated with the metabolism of toxic material in the body [28]. Inherited genetic differences in metabolism can have an effect on a population level, rather than on an individual level, and may result in different effects for a given exposure [28]. Thus, many single nucleotide polymorphisms (SNPs) result in altered expression or activity of the gene product and may modulate the response to a toxicant. Such biomarkers can also involve enzymes responsible for DNA repair and tumour suppressor proteins. These enzymes or signalling proteins are of importance for the toxicity of many toxicants and may modulate the response to mixed or combined exposures. If a certain well-characterized SNP can be shown to influence the response to a poorly defined complex mixture, it might give information regarding which components of a complex mixture are active.

## 4. The Biomonitoring Matrix

Biomarkers can be measured in exhaled air, blood, urine and in tissue samples. The actual target organ or cell is usually not available for measurements and biomarkers of exposure are thus often surrogate measures of doses or effects at the target. The ideal biomarker has been described as chemical-specific, detectable at low (trace) levels, available using non-invasive techniques, inexpensive to analyse and quantitatively related to prior exposures [30]. Thus, for biomonitoring purposes, biological materials should be easily accessible in sufficient amounts under routine conditions and without unacceptable discomfort and health risk for the individual. For these reasons blood and urine are most commonly used and cells in blood may provide surrogate endpoints for effects in internal organs [31]. Hair, teeth and nails have also been used for biomonitoring, but the knowledge of these media and biomarkers is limited [32]. New non-invasive methods, such as saliva and breath, are under development [33,34]. Sampling of exfoliated buccal cells for biomonitoring is another non-invasive technique employed, but these assays may require further improvement and validation [35]. The choice of matrix may also influence the exposure time a marker will reflect. Levels of chemicals in blood usually reflect a short time period of exposure (a few hours or days) [17] whereas adduct levels in blood proteins may reflect a much longer time of exposure.

## 5. Target Organs

### 5.1. Kidney

Heavy metals exhibit very long biological half-lives and are toxic at very low doses, and there are numerous studies on combined effects of metals. Among many organs affected by metals, the kidney is one important target organ, which relates to the kidney’s ability to reabsorb and accumulate divalent metals. The combined exposure to metals such as lead, cadmium and arsenic may lead to both additive and synergistic effects [36], but also antagonistic effects have been described. Traditional endpoints of toxicity have been morphological changes and biochemical markers for kidney toxicity and these have been shown useful at high dose exposures. Other biomarkers such as oxidative stress, altered heme biosynthesis and increases in different stress proteins could be more suitable for evaluating toxicity at lower doses [36].

In kidney, combination exposure of metals such as lead, cadmium and arsenic results in increased urinary excretion of porphyrins and this have been suggested as a good biomarker for the combined or mixed exposure of metals [17,36]. The expression of metal-binding proteins and methyl-transferase-mediated metabolic pathways may play important roles in mediating the outcomes of combined or mixed exposure to metals and can thus be regarded as good biomarkers [36]. Furthermore, metallothioneins have also been suggested as useful biomarkers for studying kidney toxicity caused by exposure to metal mixtures. Induction of metallothioneins could affect other metals by altering toxicokinetic and toxicodynamic processes. A recent example concerns urinary levels of beta-2-microglobulin and *N-*acetyl-β-D-glucosaminidase. These biomarkers of renal tubular damage were measured in a population in China exhibiting increased levels of arsenic and cadmium in urine [37]. Metal exposure gave considerably higher biomarker values than exposure to each metal alone.

Urinary excretion of enzymes such as α-glutathione-*S*-transferase has been found useful in biomonitoring of early changes in the proxitubular structure and function in occupationally exposed workers. Exposure to metal mixtures has also been shown to result in clastogenic and aneugenic effects in peripheral lymphocytes [36].

Urinary excretion of the oxidative stress marker 8-hydroxyguanine was measured in 66 nickel-cadmium battery workers [38]. A correlation between workers having high nickel concentration in urine, high cadmium concentration in blood and high levels of 8-hydroxyguanine in urine was observed.

### 5.2. Liver

Solvents may damage liver cells and liver transaminases may be used to monitor liver damage after combined or mixed exposure. In a toxicity study of a chloroform trichloroethylene mixture, alanine aminotransferase (ALT) activity in rat blood plasma was measured and combined with a histopathological assessment. In this case data suggested that the two substances had an antagonistic effect [39].

In a study of workers exposed to a mixture of solvents in car repainting shops, different biochemical parameters of liver function were measured, such as alkaline phosphatase (ALP), total bilirubin (TB) and aspartate aminotransferase (AST). These markers showed significantly higher levels in the workers compared to a control group. Furthermore, the authors found that serum bile acids were the most sensitive markers for detecting liver injury, suggesting that serum bile acids could be a valuable biomarker of hepatotoxicity caused by organic solvents [40]. The cumulative (over time) exposure of solvents and liver biomarkers was evaluated in 29 exposed workers. The study reports that higher liver enzymes activities of AST and ALT in blood related to exposure during the past 5 years, while higher levels of triglycerides reflected the total lifetime cumulative solvent exposure [41].

### 5.3. Lung

Many inhaled toxicants affect the lungs and the Clara cell protein CC16 in serum has been used as a biomarker of lung effects in studies on complex occupational exposures [42,43]. In addition, more general markers of genotoxicity such as bulky DNA adducts, oxidative stress markers, and mutations in surrogate tissues or cells have been used for measuring effects of the combined exposure to air pollutants [44,45].

### 5.4. Nervous System

The complexity of the nervous system has hindered the development of biomonitoring strategies for chemicals affecting this organ. Metals, solvents and pesticides have been measured as parent compounds or as metabolites in blood, urine and hair [46]. In particular, biomarkers of effect have been difficult to establish, which may relate to the facts that even very subtle alterations in small groups of cells can have clinically detectable effects. One example of biomonitoring the effects of relevance to the nervous system is measurements of acetylcholinesterase activity in red blood cells following complex exposure to organophosphorus insecticides [46].

### 5.5. Blood

Blood is commonly used for biomonitoring purposes. Levels of chemicals and/or their metabolites can be measured in blood. Many other types of biomarkers like micronuclei; acetylcholinesterase activity *etc.* can be measured in blood. White blood cell counts, or myelosuppression, may be used to monitor drug effects in clinical settings in cancer patients exposed to cocktails of chemotherapeutic drugs [47].

## 6. Biomonitoring a Joint Mode of Action: Oxidative Stress

Low levels of oxidative stress may reflect normal metabolism, but oxidative stress is also a common pathological process that might have a role in the development of many diseases. Inflammation and oxidative stress are involved in chronic diseases such as atherosclerosis and tissue fibroses, and are seen in many lung diseases. Another disease commonly associated with oxidative stress is cancer. Many metals including arsenic and selenium as well as many xenobiotics, such as dioxins, PCBs, PAHs and other carcinogens have been shown to cause oxidative stress. This means that monitoring oxidative stress could be an informative way to study interactions between numerous toxicants. However, it should be kept in mind that oxidative stress sometimes is an important causative factor and sometimes only a bystander in an agent’s, or a mixture’s, toxicological profile.

Endogenous DNA adducts that result from oxidative stress are always present in genomic DNA and may be generated as artefacts during sample preparation. The non-zero background causes uncertainty when risks are extrapolated from hazardous chemicals that produce oxidative stress that is important for toxicity and/or carcinogenicity. Base oxidation is one of the most frequent insults to DNA and commonly used biomonitoring markers are end products of oxidative DNA damage. These markers include e.g., 8-oxo-7,8-dihydro-2′-deoxyguanisine and malondialdehyde-dG adducts [48].

Markers of oxidative stress have been demonstrated to be sensitive effect biomarkers both at high and low doses of combined metal exposures. A common cellular target for single metal exposure is the mitochondria, which is also the major intracellular source of reactive oxygen species. Oxidative stress caused by mixtures of metals may lead to an increased malondialdehyde (a breakdown product of peroxidised fatty acids) excretion in urine. Induction of 8-hydroxy-2′-deoxy-guanosine and an up-regulated expression of antioxidant enzymes, such as superoxide dismutase and glutathione peroxidases, has also been shown after combined exposures [36].

When investigating the role of oxidative stress in the pathogenesis of disease, and when using oxidative stress as endpoint, a panel of biomarkers, specific for the different types of oxidative stress-induced damages, might be advantageous [49].

## 7. Biomarkers and Cancer

The field of biomarkers reflecting carcinogen exposure and effects is well studied. A possible reason for this interest is the knowledge that cancer development takes many years and that there is a need for early markers of effect. A latency of 10–40 years between first exposure and diagnosis is commonly anticipated. In addition, interactions between different types of carcinogens, e.g., initiators and promoters and their interactions over time have been well characterised in animal models. These circumstances have led to the development of many biomarkers of exposure and effect [50] and fortunately, many of the effect biomarkers could be useful for monitoring both combined and mixed exposures.

As mentioned above, analysis of 1-hydroxypyrene in urine has been used to monitor exposure of PAHs. It is important to note that many endpoints used in studies concerning carcinogens are not compound specific but are targets for many chemicals or groups of chemicals. Thus, they are sometimes used for biomonitoring exposures to mixtures or for biomonitoring aggregated effects.

Genotoxic xenobiotics cause direct DNA damage, which can be biomonitored as a single endpoint, although the type of DNA damage may vary. It is important, however, to keep in mind that not all DNA adducts are equally prone to cause mutations. The mutagenic capacity may depend on e.g., type of adduct, the capacity for DNA repair and cell type. Furthermore, DNA adducts are also formed endogenously, from normal metabolic or dietary components [27]. Endpoints useful for biomonitoring of complex mixtures include the so called comet assay which measure DNA strand breaks. Surrogate target cells include samples of blood lymphocytes or buccal leucocytes. The buccal leucocytes approach has been used for biomonitoring asphalt workers exposed to the complex mixture in asphalt fumes [51].

Measuring urine mutagenicity by employing salmonella tests for mutagenicity, so called Ames tests, should integrate the effects of all mutagenic compounds excreted in urine. A group of 29 workers studying or teaching in organic chemistry and exposed to organic solvents were tested for their urine mutagenicity. Mutagenicity was assayed with the salmonella plus microsome assay. Compared to controls, significant differences in mutagenic activity of urine samples were detected [52]. The authors suggest that this effect was due to the combined solvent exposure, but the sources of mutagenicity were not studied. Another study, employing several biomonitoring endpoints were selected to demonstrate effects of complex polluting mixtures, analysed blood from children in Silesia in Polen. Pollutants were derived from mining, smelting activities, heavy industry with coal-based power, steel and coke plants, heavy automobile traffic and coal for domestic heating. Among other findings, a correlation between PAH exposure, measured as 1-hydroxypyrene in urine, correlated with sister chromatid exchanges in peripheral lymphocytes [53]. Previously, adults living in the same geographical area were investigated in a seminal study in the field of biomonitoring [54]. A partially different battery of biomonitoring endpoints reflecting precarcinogenic effects were related to residency in this area, and the highest levels were often recorded in samples taken during winter. For example, overexpression of the ras oncogene in plasma was found in the samples taken during winter, suggesting a strong influence of complex exposure caused by domestic coal heating.

Mutations as biomarkers of cancer represent more specific endpoints than DNA damage. Mutations can occur in reporter genes, such as HPRT (*i.e.*, genes not related to cancer development, but used as surrogates because they are relatively easily evaluated), or more specifically, in oncogenes or tumour suppressor genes. The mutation spectra in the tumour suppressor p53 have been extensively studied. Some mutations have been claimed to reflect carcinogen specificity, such as the codon 249 mutations caused by aflatoxin exposure [55]. Many other “hot spot” mutations in the p53 gene have not been associated with single carcinogenic compounds but may reflect effects of many xenobiotics, and should be suitable for biomonitoring mixtures. Other mutations have been associated with oxidative stress and may thus reflect endogenous oxidative stress induced e.g., by inflammation or by oxidative stress inducing xenobiotics or viruses [56]. Interestingly, the detection of mutated p53 protein in blood has been correlated to aflatoxin exposure [56], but may also reflect complex carcinogenic exposures.

Other biomarkers of early effects are chromosome aberrations, micronuclei and aneuploidy. A recent example is the evaluation of markers for genotoxicity in 30 workers exposed to low doses of antineoplastic drugs and 57 workers exposed to low doses of PAHs (including 41 airport workers and 16 paving workers) [57]. Micronucleus and comet assays were performed on lymphocytes and exfoliated buccal cells. The micronucleus assays on buccal cells showed significantly higher values in workers exposed to antineoplastic drugs as compared to controls. In addition, buccal cells proved to be the best target cells for the comet assay when the biological effects of PAH mixtures were evaluated; a significant difference between PAH-exposed workers and their respective control group was found for tail moment in the comet assay [57]. It has been suggested that increased levels of micronuclei in blood could be used to predict cancer risk, especially for urogenital and gastro-intestinal cancers [58].

An example of effect biomarkers related to carcinogens was shown in a recent study where levels of chromosomal damage endpoints such as nucleoplasmic bridges and nuclear buds were significantly higher in workers exposed to PAHs compared to controls. The authors suggested that these endpoints were sensitive and reliable biomarkers for genetic damage induced by PAHs [59].

We have found no literature on general biomarkers suitable for non-mutagenic carcinogens. Enzyme induction, cell proliferation, inhibition of gap junction intracellular communication and modulation of apoptosis are examples of common modes of action for these types of carcinogens [60]. These endpoints have been mostly studied in animal and cell experiments. It can be added that a large array of signalling molecules are critical for non-mutagenic carcinogenic effects and that genomic and proteomic approaches should be well suited for the future development of this area of research. Such endpoints should help to identify novel potential biomarkers for non-mutagenic carcinogenic effects of chemicals in humans [17].

## 8. Biomarkers and Endocrine Disrupting Chemicals

Recent studies indicate that endocrine disrupting chemicals may interact in complex ways (for a comprehensive and recent overview see [61]). In particular endocrine disrupting chemicals may interact over time, in a way similar to that of many carcinogens. The endocrine disrupting mode of action has caused concern, especially as even low exposure during foetal or early life periods might be involved [62]. This characteristic will certainly complicate the detection of harmful interactions in epidemiological studies and there is a great need for reliable biomarkers of effect. Endocrine disrupters are mainly assumed to act via epigenetic effects and prospects for epigenetic epidemiology have recently been reviewed [63]. This paper summarizes environmental risk factors, exposure scenarios over time and methodology. Unfortunately, published biomonitoring data on endocrine disrupting chemicals mainly focus on agent-specific exposure markers, not on effect markers.

Phthalates are ubiquitous xenobiotics found in many plastic products, cosmetics *etc.* Many phthalates have shown endocrine disrupting and anti-androgenic properties in animal studies, but usually these phthalates have been investigated one at a time. However, in a recent study on newborn boys, their mothers were monitored during pregnancy for prenatal phthalate exposure, and several phthalates derived from domestic and life style related exposures were biomonitored [64]. The ubiquitous occurrence of phthalates suggests that analysing metabolites in urine is a way to avoid contamination problems. A combined phthalate exposure index was defined for each pregnant woman/mother and it was found that this index of combined phthalate exposure correlated to the anogenital distance (AGD). A reduction of the AGD has previously been associated with anti-androgen effects of hormonal disruption in animal studies. This study suggests that the exposure of pregnant women to phthalates in the daily environment may have effects on foetal development. However, additional studies showing similar effects of similar exposures are needed for more definitive conclusions and the effect of single phthalates *versus* the combined effect of all analysed phthalates remains to be studied. It can be noted that the index for combined exposure gave a stronger significance compared with the significance for any single phthalate, suggesting a major contribution by the combined action.

In a later study, different matrices for biomonitoring phthalate exposure were evaluated. It was found that monitoring urinary levels of phthalate metabolites was a better method for monitoring exposure than measuring metabolites in breast milk or in blood [65]. More comprehensive reviews on the biomonitoring of phthalate exposure were recently published [66,67].

Estrogenicity caused by combined or mixed exposures could be determined by using the concept of “total effective xenoestrogenic burden” (TEXB). TEXB may serve as a biomarker of endocrine disruption. Human specimens such as adipose tissue could be used to determine the combined or mixed effect of xenoestrogens using the estrogen screen (E-screen) bioassay. In this method, xenoestrogens are separated from endogenous hormones by HPLC. Fractions are then tested for estrogenic activity using the E-screen; the activity in MCF-7 human breast cancer cells treated with oestrogen (as a positive control) is compared with the activity in tissue extracts. The effect on cell proliferation is then evaluated. The effective burden represents the combined effects of chemical compounds in the tissue extract [68]. This procedure has also been applied when analysing xenoestrogenic extracts from human placenta [69], and was used in a case-control study. 50 newborn boys with cryptorchism and/or hypospadias were compared with 114 boys without malformations. 72% of the cases and 54% of the controls had detectable xenoestrogens in their placentas and this difference was significant (p < 0.05) [70]. This study support the idea that TEXB measured in placenta extract can be used as a biomarker of exposure in studies on hormonal disruption.

It has been suggested that dichloroanilines in human urine can be used as common exposure markers for several pesticides, including pesticides with endocrine disrupting properties [71]. In a recent study, the association between exposures to organophosphate compounds and serum levels of thyroid hormones were studied in floriculture workers (136 men). Serum increases of both TSH and T(4) hormones were associated with an increase in total dimethylphosphate levels in urine and a decrease in total T(3) serum level. The authors conclude that the findings support the hypothesis that organophosphate pesticides can act as endocrine disruptors in humans [72].

With the aim to shorten animal studies, effect markers for malformations have been studied in animals [73]. After *in utero* and postnatal exposure to mixtures of anti-androgens the AGD, nipple retention and a dysgenesis score were measured. It was found that at least the AGD at birth predicted later developed hypospadias. During a later rat study, the same authors used these endpoints and described a synergistic interaction between four androgen disrupters. Di(2-ethylhexyl)phthalate, vinclozolin, prochloraz and finasteride were selected for the study as they act with differing mechanisms [74].

The hypothesis of a testicular dysgenesis syndrome (TDS) is based on several observations on early manifestations at birth, such as cryptorchism and hypospadias and later development of infertility and germ cell cancer in testes of young men [75]. A role of endocrine disrupting chemicals has been discussed, and several associations have been documented in humans. As mentioned above [74], animal studies have shown that combined exposures to low doses, incapable to induce effects of their own, can result in a reduced AGD [76]. It is thus feasible that future studies on testicular cancer or infertility may use early manifestations of TDS as effect biomarkers of chemicals causing endocrine disruption.

Efforts to develop markers for endocrine-disrupting chemicals involved in prostate cancer development have been reported. It has been shown in rats that methylations of certain genes, such as PDE4D4, are induced by e.g., bisphenol A and estradiol very early in prostate cancer development and may facilitate prostate cancer development. An advantage of measuring this protein change is that it can be detected before the onset of histopathological changes. The authors suggest that this and other methylations in the genome can be used as markers for epigenetic changes induced by environmental endocrine disruptors [77]. This type of effect marker might be informative in epidemiological studies on endocrine-disrupting chemicals which include data from biopsy material.

## 9. Future Perspectives

Cancer-causing chemicals may alter gene expression by epigenetic mechanisms, and such mechanisms have the potential to become important biomarkers in future applications. Importantly, epigenetic changes may often be of reversible nature and detecting such early changes can not only be used for risk assessment purposes but also benefit both therapeutic as well as preventive monitoring [78].

For combined or mixed exposures an array of biomarkers has been suggested, where each individual biomarker provide some of the ideal characteristics of a specific biomarker [28]. For example, to accurately evaluate inhaled complex mixtures, measuring a panel of biomarkers is suggested [18]. The development of newer techniques such as gene arrays and proteomics may yield interesting results within this field. Large number of genes can be monitored and alterations in gene expression after combined exposure can be analysed [17]. The best biomarker in this sense would be a combination of approaches at different levels of cellular organization, such as DNA, RNA and proteins [79]. There is also a need for development of methods that measure effects at low dose levels [17].

The US EPA suggests that specific research is focused on using pharmacokinetic (PBPK) models to understand biomarker data and to estimate target tissue doses in cases where surrogate targets have been used. The US EPA further suggests that the biochemical and mathematical relationships among biomarkers, exposures, and internal dose for non-persistent chemicals needs to be evaluated. Linking exposure to health effects using a system biology approach is yet another future challenge [34].

Recent initiatives in changing toxicity testing, from being mainly based on animal tests to cell based techniques [80] should not only speed up the testing of old and new chemicals, but may also give valuable input in the field of biomonitoring. According to “Toxicity Testing in the 21st Century: A Vision and Strategy” [81] a key issue is to define toxicity pathways, signaling pathways that are perturbed by toxic chemicals. Estimates on how many such pathways that will be defined in the future vary from 132 to an unlimited number. Besides, this search for signaling pathways may result in the identification of molecular “nodes” in cells that are particularly vulnerable to chemical insults. Enormous resources are now put into high-throughput screening of chemicals in cell based models, and efforts are made to get proof-in-principal data showing that pathway perturbations can predict results from animal testing [82].

Ideally, alterations in a limited number of such nodes may signify key toxic events in response to large groups of chemicals. It can then be anticipated that alterations in such nodes may integrate complex signaling in several pathways. The tumour suppressor protein p53 is one of several endpoints that will be monitored in ongoing high-throughput studies [80], and alterations in this protein may reflect chemical exposures leading to DNA damage and repair. This can be induced by a large group of DNA-binding chemicals and by chemicals inducing oxidative stress and inflammation. Furthermore, p53 alterations are also induced by other stressors and may integrate e.g., DNA repair activities with nutritional status *etc.* This integration may result in cell cycle stop for DNA repair, or alternatively in replicative senescence or apoptosis. All three responses may have different pathological consequences and may affect the toxicological profile of a chemical, a chemical mixture or combined exposure.

From this on-going process we can expect a quantity of data of more or less direct importance for biomonitoring. It can e.g., be expected that some of the nodes discussed above should be close to ideal for biomonitoring purposes of complex or mixed exposures. However, critics are worried that cell signaling might be more complex than presently anticipated. For example, the database PubMed presently lists 837 papers on crosstalk between signaling pathways, indicating a layer of complexity that is often overlooked. Furthermore, uncountable papers on posttranslational modifications of gene products further increase the complexity in delineating a cell’s signaling [83].

Other potentially interesting targets for biomonitoring are telomeres. These DNA stretches form the end of chromosomes and are shortened by cell divisions, and it has been suggested that telomere attrition might be used for biomonitoring purposes [84]. Several papers have been published indicating a correlation between oxidative stress, inflammation, atherosclerosis, hypertension, chronic obstructive pulmonary disease, aging and telomere attrition. Chemically induced stresses may lead to these types of effects and indeed, smoking has repeatedly been shown to enhance telomere attrition [85]. Furthermore, telomere length may also function as a marker for susceptibility [84,86,87], suggesting that measuring telomere attrition might be an informative marker for many types of complex chemical exposures. It was recently showed that the telomere length in peripheral blood leukocytes of professionals exposed to PAHs [88] or to traffic pollution [89] was shortened. Inflammation and oxidative stress seems to be common factors underlying these effects [90]. A complicating factor is that telomerase activity may compensate for telomere shortening, in leucocytes [91].

A so far unanswered question is which cell types should be used for biomonitoring these endpoints. Toxicological processes may of course take place in many parenchymal cells in the body, not accessible for biomonitoring. It is thus clear that in most cases surrogate cell types such as blood lymphocytes have to be used. Their reliability for this purpose, as well as other indirect biomatrices, remains to be evaluated.

In parallel with the development and increased use of methods for human biomonitoring, also ethical questions arise that may need consideration. These issues may vary from sector to sector in society. Ethical issues have been discussed in several recent articles, see e.g., [6,92,93], and should be applicable regardless whether single, mixed or combined exposures are biomonitored.

In conclusion, even though we have found a number of studies evaluating the use of biomarkers and biomonitoring for combined or mixed exposures, further development in this area is urgently needed. There are many questions to answer about presently used biomarkers and their relation to health effects. Future goals include the development of specific biomarkers for combined or mixed exposure taking advantage of the ongoing characterization of toxicity signaling pathways.

## References

[1] (1986). Guidelines for the Health Risk Assessment of Chemical Mixtures.

[2] (2000). Supplementary Guidance for Conducting Health Risk Assessment of Chemical Mixtures.

[3] (2003). US-EPA Framework for Cumulative Risk Assessment.

[4] IPCS Assessment of Combined Exposures.

[5] (2009). Council Conclusions on Combination Effects of Chemicals.

[6] Manno M, Viau C, Cocker J, Colosio C, Lowry L, Mutti A, Nordberg M, Wang S (2010). Biomonitoring for occupational health risk assessment (BOHRA). Toxicol. Lett.

[7] Sexton K, Hattis D (2007). Assessing cumulative health risks from exposure to environmental mixtures—three fundamental questions. Environ. Health Perspect.

[8] Groten JP, Feron VJ, Suhnel J (2001). Toxicology of simple and complex mixtures. Trends Pharmacol. Sci.

[9] (2004). Guidance Manual for the Assessment of Joint Toxic Action of Chemical Mixtures.

[10] Cassee FR, Groten JP, van Bladeren PJ, Feron VJ (1998). Toxicological evaluation and risk assessment of chemical mixtures. Crit. Rev. Toxicol.

[11] Mumtaz MM, Ruiz P, De Rosa CT (2007). Toxicity assessment of unintentional exposure to multiple chemicals. Toxicol. Appl. Pharmacol.

[12] Mumtaz MM, Tully DB, El-Masri HA, De Rosa CT (2002). Gene induction studies and toxicity of chemical mixtures. Environ. Health Perspect.

[13] Viau C (2002). Biological monitoring of exposure to mixtures. Toxicol. Lett.

[14] Spurgeon DJ, Jones OA, Dorne JL, Svendsen C, Swain S, Sturzenbaum SR (2010). Systems toxicology approaches for understanding the joint effects of environmental chemical mixtures. Sci. Total Environ.

[15] Kortenkamp A, Backhaus T, Faust M (2009). State of the Art Report on Mixture Toxicity.

[16] Jenkins S, Raghuraman N, Eltoum I, Carpenter M, Russo J, Lamartiniere CA (2009). Oral exposure to bisphenol a increases dimethylbenzanthracene-induced mammary cancer in rats. Environ. Health Perspect.

[17] Watson WP, Mutti A (2004). Role of biomarkers in monitoring exposures to chemicals: Present position, future prospects. Biomarkers.

[18] Scherer G (2005). Biomonitoring of inhaled complex mixtures—ambient air, diesel exhaust and cigarette smoke. Exp. Toxicol. Pathol.

[19] Guha N, Steenland NK, Merletti F, Altieri A, Cogliano V, Straif K (2010). Bladder cancer risk in painters: a meta-analysis. Occup. Environ. Med.

[20] (2009). A Review of Human Carcinogens—Part F.

[21] Bergvall C, Westerholm R (2007). Identification and determination of highly carcinogenic dibenzopyrene isomers in air particulate samples from a street canyon, a rooftop, and a subway station in Stockholm. Environ. Sci. Technol.

[22] Alexandrie AK, Warholm M, Carstensen U, Axmon A, Hagmar L, Levin JO, Ostman C, Rannug A (2000). CYP1A1 and GSTM1 polymorphisms affect urinary 1-hydroxypyrene levels after PAH exposure. Carcinogenesis.

[23] Elovaara E, Heikkila P, Pyy L, Mutanen P, Riihimaki V (1995). Significance of dermal and respiratory uptake in creosote workers: exposure to polycyclic aromatic hydrocarbons and urinary excretion of 1-hydroxypyrene. Occup. Environ. Med.

[24] McClean MD, Rinehart RD, Ngo L, Eisen EA, Kelsey KT, Wiencke JK, Herrick RF (2004). Urinary 1-hydroxypyrene and polycyclic aromatic hydrocarbon exposure among asphalt paving workers. Ann. Occup. Hyg.

[25] Seidel A, Spickenheuer A, Straif K, Rihs HP, Marczynski B, Scherenberg M, Dettbarn G, Angerer J, Wilhelm M, Bruning T, Jacob J, Pesch B (2008). New biomarkers of occupational exposure to polycyclic aromatic hydrocarbons. J. Toxicol. Environ. Health A.

[26] Caro J, Gallego M (2009). Environmental and biological monitoring of volatile organic compounds in the workplace. Chemosphere.

[27] Jarabek AM, Pottenger LH, Andrews LS, Casciano D, Embry MR, Kim JH, Preston RJ, Reddy MV, Schoeny R, Shuker D, Skare J, Swenberg J, Williams GM, Zeiger E (2009). Creating context for the use of DNA adduct data in cancer risk assessment: I. Data organization. Crit. Rev. Toxicol.

[28] Ryan PB, Burke TA, Cohen Hubal EA, Cura JJ, McKone TE (2007). Using biomarkers to inform cumulative risk assessment. Environ. Health Perspect.

[29] Mahadevan B, Keshava C, Musafia-Jeknic T, Pecaj A, Weston A, Baird WM (2005). Altered gene expression patterns in MCF-7 cells induced by the urban dust particulate complex mixture standard reference material 1649a. Cancer Res.

[30] Henderson RF, Bechtold WE, Bond JA, Sun JD (1989). The use of biological markers in toxicology. Crit. Rev. Toxicol.

[31] Angerer J, Ewers U, Wilhelm M (2007). Human biomonitoring: state of the art. Int. J. Hyg. Environ. Health.

[32] Esteban M, Castano A (2009). Non-invasive matrices in human biomonitoring: A review. Environ. Int.

[33] Amorim LC, de L. Cardeal Z (2007). Breath air analysis and its use as a biomarker in biological monitoring of occupational and environmental exposure to chemical agents. J. Chromatogr. B Analyt. Technol. Biomed. Life Sci.

[34] USEPA Biomarkers for Cumulative Risk Assessment.

[35] Ceppi M, Biasotti B, Fenech M, Bonassi S (2010). Human population studies with the exfoliated buccal micronucleus assay: Statistical and epidemiological issues. Mutat. Res.

[36] Wang G, Fowler BA (2008). Roles of biomarkers in evaluating interactions among mixtures of lead, cadmium and arsenic. Toxicol. Appl. Pharmacol.

[37] Nordberg GF (2010). Biomarkers of exposure, effects and susceptibility in humans and their application in studies of interactions among metals in China. Toxicol. Lett.

[38] Yoshioka N, Nakashima H, Hosoda K, Eitaki Y, Shimada N, Omae K (2008). Urinary excretion of an oxidative stress marker, 8-hydroxyguanine (8-OH-Gua), among nickel-cadmium battery workers. J. Occup. Health.

[39] Anand SS, Mumtaz MM, Mehendale HM (2005). Dose-dependent liver tissue repair after chloroform plus trichloroethylene binary mixture. Basic Clin. Pharmacol. Toxicol.

[40] Nunes de Paiva MJ, Pereira Bastos de Siqueira ME (2005). Increased serum bile acids as a possible biomarker of hepatotoxicity in Brazilian workers exposed to solvents in car repainting shops. Biomarkers.

[41] Kaukiainen A, Vehmas T, Rantala K, Nurminen M, Martikainen R, Taskinen H (2004). Results of common laboratory tests in solvent-exposed workers. Int. Arch. Occup. Environ. Health.

[42] Backe E, Lotz G, Tittelbach U, Plitzko S, Gierke E, Schneider WD (2004). Immunological biomarkers in salt miners exposed to salt dust, diesel exhaust and nitrogen oxides. Int. Arch. Occup. Environ. Health.

[43] Ulvestad B, Randem BG, Andersson L, Ellingsen DG, Barregard L (2007). Clara cell protein as a biomarker for lung epithelial injury in asphalt workers. J. Occup. Environ. Med.

[44] Avila Junior S, Possamai FP, Budni P, Backes P, Parisotto EB, Rizelio VM, Torres MA, Colepicolo P, Wilhelm Filho D (2009). Occupational airborne contamination in south Brazil: 1. Oxidative stress detected in the blood of coal miners. Ecotoxicology.

[45] Vineis P, Husgafvel-Pursiainen K (2005). Air pollution and cancer: biomarker studies in human populations. Carcinogenesis.

[46] Costa LG (1996). Biomarker research in neurotoxicology: the role of mechanistic studies to bridge the gap between the laboratory and epidemiological investigations. Environ. Health Perspect.

[47] Yano Y, Kodawara T, Hongo H, Yano I, Kishi Y, Takahashi J, Inui K (2009). Population analysis of myelosuppression profiles using routine clinical data after the ICE (ifosfamide/carboplatin/etoposide) regimen for malignant gliomas. J. Pharm. Sci.

[48] Taioli E, Sram RJ, Binkova B, Kalina I, Popov TA, Garte S, Farmer PB (2007). Biomarkers of exposure to carcinogenic PAHs and their relationship with environmental factors. Mutat. Res.

[49] Swenberg JA, Fryar-Tita E, Jeong YC, Boysen G, Starr T, Walker VE, Albertini RJ (2008). Biomarkers in toxicology and risk assessment: informing critical dose-response relationships. Chem. Res. Toxicol.

[50] Dunn BK, Wagner PD, Anderson D, Greenwald P (2010). Molecular markers for early detection. Semin. Oncol.

[51] Lindberg HK, Vaananen V, Jarventaus H, Suhonen S, Nygren J, Hameila M, Valtonen J, Heikkila P, Norppa H (2008). Genotoxic effects of fumes from asphalt modified with waste plastic and tall oil pitch. Mutat. Res.

[52] Varella SD, Rampazo RA, Varanda EA (2008). Urinary mutagenicity in chemical laboratory workers exposed to solvents. J. Occup. Health.

[53] Mielzynska D, Siwinska E, Kapka L, Szyfter K, Knudsen LE, Merlo DF (2006). The influence of environmental exposure to complex mixtures including PAHs and lead on genotoxic effects in children living in Upper Silesia, Poland. Mutagenesis.

[54] Perera FP, Hemminki K, Gryzbowska E, Motykiewicz G, Michalska J, Santella RM, Young TL, Dickey C, Brandt-Rauf P, De Vivo I (1992). Molecular and genetic damage in humans from environmental pollution in Poland. Nature.

[55] Besaratinia A, Kim SI, Hainaut P, Pfeifer GP (2009). *In vitro* recapitulating of TP53 mutagenesis in hepatocellular carcinoma associated with dietary aflatoxin B1 exposure. Gastroenterology.

[56] Hussain SP, Schwank J, Staib F, Wang XW, Harris CC (2007). TP53 mutations and hepatocellular carcinoma: insights into the etiology and pathogenesis of liver cancer. Oncogene.

[57] Cavallo D, Ursini CL, Rondinone B, Iavicoli S (2009). Evaluation of a suitable DNA damage biomarker for human biomonitoring of exposed workers. Environ. Mol. Mutagen.

[58] Bonassi S, Znaor A, Ceppi M, Lando C, Chang WP, Holland N, Kirsch-Volders M, Zeiger E, Ban S, Barale R, Bigatti MP, Bolognesi C, Cebulska-Wasilewska A, Fabianova E, Fucic A, Hagmar L, Joksic G, Martelli A, Migliore L, Mirkova E, Scarfi MR, Zijno A, Norppa H, Fenech M (2007). An increased micronucleus frequency in peripheral blood lymphocytes predicts the risk of cancer in humans. Carcinogenesis.

[59] Duan H, Leng S, Pan Z, Dai Y, Niu Y, Huang C, Bin P, Wang Y, Liu Q, Chen W, Zheng Y (2009). Biomarkers measured by cytokinesis-block micronucleus cytome assay for evaluating genetic damages induced by polycyclic aromatic hydrocarbons. Mutat. Res.

[60] Hattis D, Chu M, Rahmioglu N, Goble R, Verma P, Hartman K, Kozlak M (2009). A preliminary operational classification system for nonmutagenic modes of action for carcinogenesis. Crit. Rev. Toxicol.

[61] Diamanti-Kandarakis E, Bourguignon JP, Giudice LC, Hauser R, Prins GS, Soto AM, Zoeller RT, Gore AC (2009). Endocrine-disrupting chemicals: an Endocrine Society scientific statement. Endocr. Rev.

[62] Soto AM, Sonnenschein C (2010). Environmental causes of cancer: endocrine disruptors as carcinogens. Nat. Rev. Endocrinol.

[63] Foley DL, Craig JM, Morley R, Olsson CA, Dwyer T, Smith K, Saffery R (2009). Prospects for epigenetic epidemiology. Am. J. Epidemiol.

[64] Swan SH, Main KM, Liu F, Stewart SL, Kruse RL, Calafat AM, Mao CS, Redmon JB, Ternand CL, Sullivan S, Teague JL (2005). Decrease in anogenital distance among male infants with prenatal phthalate exposure. Environ. Health Perspect.

[65] Hogberg J, Hanberg A, Berglund M, Skerfving S, Remberger M, Calafat AM, Filipsson AF, Jansson B, Johansson N, Appelgren M, Hakansson H (2008). Phthalate diesters and their metabolites in human breast milk, blood or serum, and urine as biomarkers of exposure in vulnerable populations. Environ. Health Perspect.

[66] Kamrin MA (2009). Phthalate risks, phthalate regulation, and public health: A review. J. Toxicol. Environ. Health B Crit. Rev.

[67] Koch HM, Calafat AM (2009). Human body burdens of chemicals used in plastic manufacture. Philos. Trans. R. Soc. Lond. B Biol. Sci.

[68] Fernandez MF, Aguilar-Garduno C, Molina-Molina JM, Arrebola JP, Olea N (2008). The total effective xenoestrogen burden, a biomarker of exposure to xenoestrogen mixtures, is predicted by the (anti)estrogenicity of its components. Reprod. Toxicol.

[69] Lopez-Espinosa MJ, Silva E, Granada A, Molina-Molina JM, Fernandez MF, Aguilar-Garduno C, Olea-Serrano F, Kortenkamp A, Olea N (2009). Assessment of the total effective xenoestrogen burden in extracts of human placentas. Biomarkers.

[70] Fernandez MF, Olmos B, Granada A, Lopez-Espinosa MJ, Molina-Molina JM, Fernandez JM, Cruz M, Olea-Serrano F, Olea N (2007). Human exposure to endocrine-disrupting chemicals and prenatal risk factors for cryptorchidism and hypospadias: A nested case-control study. Environ. Health Perspect.

[71] Wittke K, Hajimiragha H, Dunemann L, Begerow J (2001). Determination of dichloroanilines in human urine by GC-MS, GC-MS-MS, and GC-ECD as markers of low-level pesticide exposure. J. Chromatogr. B Biomed. Sci. Appl.

[72] Lacasana M, Lopez-Flores I, Rodriguez-Barranco M, Aguilar-Garduno C, Blanco-Munoz J, Perez-Mendez O, Gamboa R, Bassol S, Cebrian ME (2010). Association between organophosphate pesticides exposure and thyroid hormones in floriculture workers. Toxicol. Appl. Pharmacol.

[73] Christiansen S, Scholze M, Axelstad M, Boberg J, Kortenkamp A, Hass U (2008). Combined exposure to anti-androgens causes markedly increased frequencies of hypospadias in the rat. Int. J. Androl.

[74] Christiansen S, Scholze M, Dalgaard M, Vinggaard AM, Axelstad M, Kortenkamp A, Hass U (2009). Synergistic disruption of external male sex organ development by a mixture of four antiandrogens. Environ. Health Perspect.

[75] Main KM, Skakkebaek NE, Virtanen HE, Toppari J (2010). Genital anomalies in boys and the environment. Best Pract. Res. Clin. Endocrinol. Metab.

[76] Sharpe RM, Skakkebaek NE (2008). Testicular dysgenesis syndrome: mechanistic insights and potential new downstream effects. Fertil. Steril.

[77] Ho SM, Tang WY, Belmonte de Frausto J, Prins GS (2006). Developmental exposure to estradiol and bisphenol A increases susceptibility to prostate carcinogenesis and epigenetically regulates phosphodiesterase type 4 variant 4. Cancer Res.

[78] Wild CP (2009). Environmental exposure measurement in cancer epidemiology. Mutagenesis.

[79] Preston RJ (2005). Mechanistic data and cancer risk assessment: the need for quantitative molecular endpoints. Environ. Mol. Mutagen.

[80] Laetz CA, Baldwin DH, Collier TK, Hebert V, Stark JD, Scholz NL (2009). The synergistic toxicity of pesticide mixtures: implications for risk assessment and the conservation of endangered Pacific salmon. Environ. Health Perspect.

[81] Committee on Toxicity Testing and Assessment of Environmental Agents (2007). Toxicity Testing in the 21st Century: A Vision and a Strategy.

[82] Martin MT, Judson RS, Reif DM, Kavlock RJ, Dix DJ (2009). Profiling chemicals based on chronic toxicity results from the U.S. EPA ToxRef Database. Environ. Health Perspect.

[83] Hainaut P, Wiman KG (2009). 30 years and a long way into p53 research. Lancet Oncol.

[84] Valdes AM, Andrew T, Gardner JP, Kimura M, Oelsner E, Cherkas LF, Aviv A, Spector TD (2005). Obesity, cigarette smoking, and telomere length in women. Lancet.

[85] Morla M, Busquets X, Pons J, Sauleda J, MacNee W, Agusti AG (2006). Telomere shortening in smokers with and without COPD. Eur. Respir. J.

[86] Broberg K, Bjork J, Paulsson K, Hoglund M, Albin M (2005). Constitutional short telomeres are strong genetic susceptibility markers for bladder cancer. Carcinogenesis.

[87] Rafnar T, Sulem P, Stacey SN, Geller F, Gudmundsson J, Sigurdsson A, Jakobsdottir M, Helgadottir H, Thorlacius S, Aben KK (2009). Sequence variants at the TERT-CLPTM1L locus associate with many cancer types. Nat. Genet.

[88] Pavanello S, Pesatori AC, Dioni L, Hoxha M, Bollati V, Siwinska E, Mielzynska D, Bolognesi C, Bertazzi PA, Baccarelli A (2010). Shorter telomere length in peripheral blood lymphocytes of workers exposed to polycyclic aromatic hydrocarbons. Carcinogenesis.

[89] Hoxha M, Dioni L, Bonzini M, Pesatori AC, Fustinoni S, Cavallo D, Carugno M, Albetti B, Marinelli B, Schwartz J, Bertazzi PA, Baccarelli A (2009). Association between leukocyte telomere shortening and exposure to traffic pollution: A cross-sectional study on traffic officers and indoor office workers. Environ. Health.

[90] Houben JM, Moonen HJ, van Schooten FJ, Hageman GJ (2008). Telomere length assessment: Biomarker of chronic oxidative stress?. Free Radic. Biol. Med.

[91] Epel ES, Lin J, Dhabhar FS, Wolkowitz OM, Puterman E, Karan L, Blackburn EH (2010). Dynamics of telomerase activity in response to acute psychological stress. Brain. Behav. Immun.

[92] Viau C (2005). Biomonitoring in occupational health: Scientific, socio-ethical, and regulatory issues. Toxicol. Appl. Pharmacol.

[93] Bauer S (2008). Societal and ethical issues in human biomonitoring—A view from science studies. Environ. Health.

